# A Dynamical Model for Activating and Silencing the Mitotic Checkpoint

**DOI:** 10.1038/s41598-017-04218-2

**Published:** 2017-06-20

**Authors:** Richard Henze, Peter Dittrich, Bashar Ibrahim

**Affiliations:** 1 Department of Mathematics and Computer Science, Friedrich Schiller University Jena, 07743 Jena, Germany; 2 Institute for Numerical Simulation, University of Bonn, 53115 Bonn, Germany

## Abstract

The spindle assembly checkpoint (SAC) is an evolutionarily conserved mechanism, exclusively sensitive to the states of kinetochores attached to microtubules. During metaphase, the anaphase-promoting complex/cyclosome (APC/C) is inhibited by the SAC but it rapidly switches to its active form following proper attachment of the final spindle. It had been thought that APC/C activity is an all-or-nothing response, but recent findings have demonstrated that it switches steadily. In this study, we develop a detailed mathematical model that considers all 92 human kinetochores and all major proteins involved in SAC activation and silencing. We perform deterministic and spatially-stochastic simulations and find that certain spatial properties do not play significant roles. Furthermore, we show that our model is consistent with *in*-*vitro* mutation experiments of crucial proteins as well as the recently-suggested rheostat switch behavior, measured by Securin or CyclinB concentration. Considering an autocatalytic feedback loop leads to an all-or-nothing toggle switch in the underlying core components, while the output signal of the SAC still behaves like a rheostat switch. The results of this study support the hypothesis that the SAC signal varies with increasing number of attached kinetochores, even though it might still contain toggle switches in some of its components.

## Introduction

Correct DNA segregation is a fundamental process during mitosis that relies on amphitelic attachment between chromosomes, principally through kinetochores and spindle microtubules. Failures during segregation lead to many human health problems, most notably aneuploidy and cancer^1, 2^. To avoid this, eukaryotic cells have evolved a surveillance control system called the spindle assembly checkpoint (SAC; ref. 3) to check whether all chromosomes are correctly attached or not. When the SAC is activated, the checkpoint system acts on the ubiquitin ligase anaphase-promoting complex/cyclosome (APC/C), presumably by sequestering the APC/C-activator Cdc20 via the mitotic checkpoint complex (MCC). If a single kinetochore stays unattached, whether naturally or artificially, the SAC stays active and can block the function of the APC/C for several hours^4^. Reddy *et al*. demonstrated that UbcH10 targets and ubiquitinates Cdc20 located in an APC/C:MCC complex^5^, decreasing its affinity to Mad2, and resulting in the repulsion of Mad2 from the MCC. The remaining complex BCC still inhibits the APC/C but is less stable than the MCC and dissociates faster (cf. Table S1). Dynein on the attached kinetochores releases p31^comet^ ^6, 7^, which lowers the concentration threshold necessary for UbcH10 to become active^5^. In this way, p31^comet^ and UbcH10 collaborate to silence the SAC.

**Figure 1:**
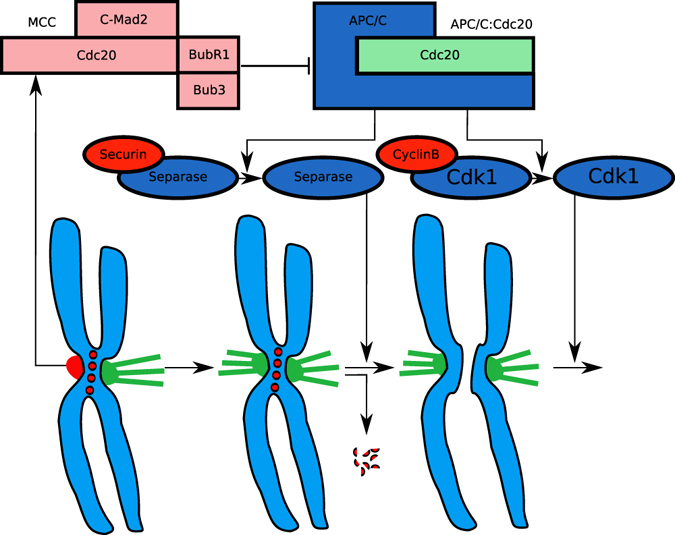
Schematic illustration of SAC activity. Unattached kinetochores (red in the middle of the chromosomes) catalyze the formation of MCC which inhibits the Cdc20-activated APC/C. Once all kinetochores are attached (green) the APC/C ubiquitinates Securin and CyclinB, resulting in the destruction of Cohesin by Separase. Unblocked Cdk1 initiates metaphase and progress in the cell cycle.

Active APC/C recruits Cdc20 and ubiquitinates Securin and CyclinB^8^. Securin inhibits Separase, a protease that is required to cleave Cohesin, which physically connects the two sister-chromatids of every chromosome^9^. Ubiquitinated CyclinB releases Cdk1, initiating mitotic exit (cf. Fig. 1).

This switch from inactive to active APC/C was believed to be an all-or-nothing response, meaning that once all kinetochores are attached APC/C starts degrading Securin and CyclinB. However, Collin *et al*. have recently shown that this is not the case^10^. They postulate that the APC/C inhibitor MCC is active at different levels, depending on the number of unattached kinetochores, resulting in an active APC/C during metaphase. This hypothesis was underpinned by the concentration curve of CyclinB, which decreased continuously depending on the MCC level. Dick *et al*. make the same claim based on Securin experiments^11^.

Computational modeling is a very important tool for elucidating how elaborate systems like the SAC work and for gaining deeper insights. Several models trying to mimic the reported behavior of the SAC have emerged. Doncic, Sear and colleagues analyzed less-detailed spatial models of potential checkpoint mechanisms with a focus on yeast and animals^12, 13^. Mistry *et al*. provided a framework accounting for the correction mechanism for improper chromosome attachment^14^. Lohel *et al*. considered models that take into account species localization and realistic kinetochore-binding kinetics, focusing on diffusion effects^15^. Models by Ibrahim and colleagues provide detailed descriptions of human SAC activation but lack a realistic explanation for the switch^16–18^. They use a manually-activated switch that enables or disables certain reactions according to one of two phases (active and silenced SAC).

None of the models mentioned so far contains a reliable mechanism for silencing the checkpoint, and all lack realistic spatial properties. Furthermore, to capture the rheostat behavior reported by^10, 11^, the respective SAC output signals, Securin and CyclinB, need to be included.

Chen *et al*. provided a compartmentalized model that gives a spatial explanation for the silencing of the SAC^19^. They account for the rheostat switch through active protein transport and their model gives a reliable explanation how SAC proteins are transported from the kinetochore region to the centrosomes. However, they emphasize that they did not focus on realistic biochemical pathways for SAC activation.

In this paper, we build the first detailed biochemical model of SAC activation and silencing that includes all essential SAC components and an output signal equivalent to Securin/CyclinB concentration. The autocatalytic feedback loop leading to SAC silencing has not been well-studied qualitatively, compared to SAC activation, and for this reason, we perform dynamical simulations at three levels of detail: full-detail spatial particle simulations including all 92 kinetochores, a non-spatial differential equation simulation, and a coarse-grained differential equation simulation extended by the putative autocatalytic feedback loop. We compare our simulations with deletion and over-expression experiments in the literature as well as with recent experiments in which Securin levels are measured for different perturbation strengths. With this, our model can be regarded as a completion of the model proposed by Chen *et al*., in that we provide a realistic biochemical pathway but do not take active transport into account.

## Results and Discussion

### Mathematical framework

**Figure 2:**
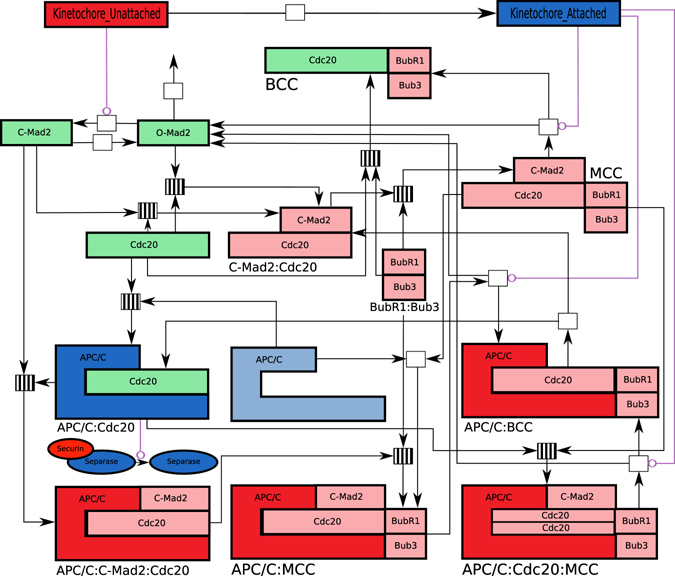
The reaction network in our model. The names of the basic species are shown in colored boxes. Molecule names are below the corresponding complexes, which are composed of basic building blocks. White boxes represent irreversible reactions, and striped boxes represent reversible reactions. In both cases, ingoing arrows are educts and outgoing arrows are products (of the corresponding forward reactions). Red boxes contain species with inhibitory functions, blue boxes contain species that promote and initiate anaphase, and green boxes contain species with non-strict functions. Purple lines show catalytic effects on the corresponding reaction.

Our approach connects two modules, one for SAC activation and one for silencing. The model consists of 17 biochemical reactions, some of which are reversible, describing the dynamics of 16 species (cf. Fig. 2). The attachment process is modeled by the reaction

1 $${{\rm{Kin}}}_{{\rm{U}}}\mathop{\rightharpoonup }\limits^{{k}_{attach}}{{\rm{Kin}}}_{{\rm{A}}}$$

where Kin_U_ and Kin_A_ denote the numbers of unattached and attached kinetochores, respectively. The next section introduces all reactions from the two modules in detail.

#### Activating the SAC

Cdc20 activated APC/C ubiquitinates Securin and CyclinB, resulting in the degradation of the latter.

2 $${\rm{APC}}/{\rm{C}}+{\rm{Cdc20}}\underset{{k}_{-1}}{\overset{{k}_{1}}{\rightleftharpoons }}{\rm{APC}}/C:\text{Cdc20}$$

3 $${\rm{APC}}/C:\text{Cdc20}+{\rm{Securin}}\mathop{\rightharpoonup }\limits^{{k}_{D}}{\rm{APC}}/C:\text{Cdc20}$$

4 $${\rm{APC}}/C:\text{Cdc20}+{\rm{CyclinB}}\mathop{\rightharpoonup }\limits^{{k}_{D}}{\rm{APC}}/C:\text{Cdc20}$$

Thus, unhindered activation of APC/C leads to the transition into anaphase. Note that we only consider Securin as a SAC output signal as both CyclinB and Securin are degraded by active APC/C, and without further information, both would have the same qualitative behavior in the simulation.

The SAC controls APC/C activity mainly via the mitotic checkpoint complex (MCC; C-Mad2:Cdc20:BubR1:Bub3), as one of its subunits (Mad2) competes with APC/C for Cdc20^20^. APC/C and MCC associate in a way that prevents Cdc20 from interacting with mitotic APC/C.

5 $${\rm{APC}}/{\rm{C}}+{\rm{MCC}}\underset{{k}_{-2}}{\overset{{k}_{2}}{\rightleftharpoons }}{\rm{APC}}/C:\text{MCC}$$

The MCC is a compound of Cdc20, Mad2 and the BubR1:Bub3 complex. It is formed in a stepwise process, though it is still not known where the assembly takes place. Regardless, the first step requires unattached kinetochore Mad1:Mad2 binding sites to catalyze the turnover from O-Mad2 to C-Mad2.

6 $$O{\rm{-}}\text{Mad2}\underset{{k}_{-3}}{\overset{k3\times [KinU]}{\rightleftharpoons }}C{\rm{-}}\text{Mad2}$$

7 $${\rm{Cdc20}}+C{\rm{-}}\text{Mad2}\mathop{\rightharpoonup }\limits^{{k}_{4}}\text{Cdc20}:C{\rm{-}}\text{Mad2}$$

8 $$C{\rm{-}}\text{Mad2}:\text{Cdc20}+\text{BubR1}:\text{Bub3}\underset{{k}_{-5}}{\overset{{k}_{5}}{\rightleftharpoons }}{\rm{MCC}}$$

As the kinetochore-mediated Mad2 turnover is rather slow, DeAntoni suggested the “Template Model”^21^, which also allows the turnover to be catalyzed by Cdc20:C-Mad2.

9 $$\text{Mad1}:\text{Mad2}+O{\rm{-}}\text{Mad2}\underset{{k}_{-T1}}{\overset{{k}_{T1}}{\rightleftharpoons }}\text{Mad1}:\text{Mad2}:C{\rm{-}}\text{Mad2}$$

10 $$\text{Mad1}:\text{Mad2}:C{\rm{-}}\text{Mad2}+{\rm{Cdc20}}\mathop{\rightharpoonup }\limits^{{k}_{T2}}\text{Mad1}:\text{Mad2}+\text{Cdc20}:C{\rm{-}}\text{Mad2}$$

As shown in previous studies^17^, the effect of these reactions is minor and, for the sake of simplicity, they are omitted from our model. Mad2 in its open conformation can also bind Cdc20 directly, although at a significantly slower rate (cf. Table S1).

11 $$O{\rm{-}}\text{Mad2}+{\rm{Cdc20}}\underset{{k}_{-6}}{\overset{{k}_{6}}{\rightleftharpoons }}C{\rm{-}}\text{Mad2}:\text{Cdc20}$$

Additionally, Cdc20 can bind BubR1:Bub3 directly to form BCC (Cdc20:BubR1:Bub3).

12 $${\rm{Cdc20}}+\text{BubR1}:\text{Bub3}\underset{{k}_{-7}}{\overset{{k}_{7}}{\rightleftharpoons }}{\rm{BCC}}$$

Howell *et al*. stated that Mad2 moves along the microtubules^22^, making the amount of Mad2 a limiting factor in the inhibition process during advanced metaphase. In our approach, this is modeled by the spontaneous decay of Mad2. However, this is not realistic as Mad2 moves towards the spindles and does not decay. This has been studied extensively by Chen *et al*.^19^ and is omitted here for the sake of simplicity. In the following expression, ∅ refers to the empty set.

13 $${\rm{Mad2}}\mathop{\rightharpoonup }\limits^{{k}_{attach}}\varnothing $$

To overcome this limitation in the inhibition process, Han and colleagues suggested an additional pathway for deactivating APC/C^23^. They introduced the formation of MCC directly at the APC/C by C-Mad2 targeting APC/C:Cdc20, which allows Cdc20 to bind the inhibiting BubR1:Bub3 complex and form MCC at the APC/C.

14 $${\rm{APC}}/C:\text{Cdc20}+C{\rm{-}}\text{Mad2}\underset{{k}_{-4}}{\overset{{k}_{4}}{\rightleftharpoons }}{\rm{APC}}/C:\text{Cdc20}:C{\rm{-}}\text{Mad2}$$

15 $${\rm{APC}}/C:\text{Cdc20}:C{\rm{-}}\text{Mad2}+\text{BubR1}:\text{Bub3}\underset{{k}_{-5}}{\overset{{k}_{5}}{\rightleftharpoons }}{\rm{APC}}/C:\text{MCC}$$

Once BubR1:Bub3 is recruited to the APC/C, Mad2 dissociates from the complex and is able to form another MCC (originally every Mad2 molecule was only able to inhibit exactly one APC/C as part of the MCC). The inhibiting level of the APC/C:BCC complex is not as high as that of APC/C:MCC, as MCC is more stable than BCC (cf. Table S1 and refs 10, 23).

As Cdc20 is the most abundant species in the system, it is not completely bound. This is not desirable because free Cdc20 can immediately activate freed APC/C. Recently, Izawa *et al*. found that the MCC binds a second Cdc20 that has already activated an APC/C^24^.

16 $${\rm{APC}}/C:\text{Cdc20}+{\rm{MCC}}\underset{{k}_{-2}}{\overset{{k}_{2}}{\rightleftharpoons }}{\rm{APC}}/C:\text{Cdc20}:\text{MCC}$$

With this reaction, the concentration of free Cdc20 is close to zero during metaphase. Note that the model for SAC activation is mostly in line with previously published models on APC/C regulation^18^.

#### Silencing the SAC

The mechanisms governing SAC silencing are still not completely understood. However, they must have the following properties to reactivate APC/C quickly: (1) extinction of the MCC fabric, namely the Mad1:Mad2 template located on unattached kinetochores, (2) disassembly of the APC/C:MCC, and (3) fast targeting of APC/C by freed Cdc20.

It has been postulated that dynein promotes pulling RZZ-Spindly-Mad1:Mad2 complexes away from outer kinetochores along microtubules^25^. Thus, once a kinetochore is attached, it is unable to turn over Mad2 from its open to closed conformation.

Released p31^comet^ is the crucial silencing factor and its amount is proportional to the number of attached kinetochores. Although technically APC/Cs act to reject Mad2, the strength of this process is dependent on the amount of p31^comet^. Therefore, attached kinetochores catalyze the removal of Mad2 from APC/C:MCC and APC/C:Cdc20:MCC in our model.

Notably, MCC already contains the activator Cdc20 and this means that dissolved MCC results in direct activation of APC/C.

17 $${\rm{APC}}/C:\text{MCC}\mathop{\rightharpoonup }\limits^{{k}_{8}\times [KinA]}{\rm{APC}}/C:\text{BCC}+O{\rm{-}}\text{Mad2}$$

18 $${\rm{APC}}/C:\text{Cdc20}:\text{MCC}\mathop{\rightharpoonup }\limits^{{k}_{9}\times [KinA]}{\rm{APC}}/C:\text{BCC}+O{\rm{-}}\text{Mad2}$$

19 $${\rm{APC}}/C:\text{BCC}\mathop{\rightharpoonup }\limits^{{k}_{10}}{\rm{APC}}/C:\text{Cdc20}+\text{BubR1}:\text{Bub3}$$

The silencing process starts with the first attached kinetochore, counteracting the formation of MCC. After its formation in an initial burst, MCC degrades continuously during metaphase, resulting in the formation of a small fraction of APC/C:Cdc20. However, activated APC/C starts degrading Securin, which explains the rheostat nature of the switch. The Securin level decreases continuously once the attachment process started, excluding the theory of an all-or-nothing switch.

In summary: (1) In an early state of metaphase, most APC/Cs are inhibited by the MCC. (2) Attached kinetochores start dissembling the stable APC/C:MCC and APC/C:Cdc20:MCC complexes by stripping away Mad2 via p31^comet^ and UbcH10 (controlled by rate constants *k*_8_ and *k*_9_). (3) During advanced metaphase, APC/C:MCC yields the less stable APC/C:BCC complex. (4) After the last attachment, APC/C is fast-reactivated as APC/C:BCC decays quickly and is no longer being formed.

The full SAC framework is depicted in Fig. 2, which includes all 17 species introduced above, and their reactions.

### ODE simulation generates wild-type behavior

As far as possible, we have used reaction parameters from the literature and previously performed *in*-*vitro* parameter studies (as listed in Table S2). Newly introduced reactions underwent a parameter study (cf. Fig. S1). The most crucial parameter turn out to be the kinetochore-allocated turnover from O-Mad2 to C-Mad2. Considering cell size and diffusion parameters, this rate was determined to be a maximal 0.016*s* ^−1^ on every single kinetochore (cf. Text S1) corresponding to a half-time of around 30 seconds. This is consistent with the experimental findings of Howell *et al*.^22^. Another crucial parameter is the kinetochore-allocated striping of Mad from APC/C:MCC complexes. Our parameter study shows that this rate should not exceed 0.015*s* ^−1^ to guarantee a functional SAC. This result will be verified later using the coarse-grained SAC model. All other parameters can be varied over a wide range (see the references for the relevant parameter studies). This is consistent with Gutenkunst *et al*.^26^, who stated that in most biological systems, qualitative behavior is not governed by parameters but by the structure of the relevant network. Here, the switch is a consequence of network topology rather than parameter values.

In our simulation of the ODE model, APC/C:MCC and APC/C:Cdc20:MCC are produced quickly and disable the ability of APC/C to ubiquitinate. Both species decrease steadily and yield the less stable APC/C:BCC complex. MCC and Cdc20:C-Mad2 levels decrease permanently, strengthening the hypothesis of Collin *et al*. that the SAC works like a rheostat switch. The concentration of free Cdc20 during the attachment process drops close to zero. Contradicting the all-or-nothing response, the level of APC/C:Cdc20 rises steadily until the final attachment, degrading Securin continuously.

**Figure 3:**
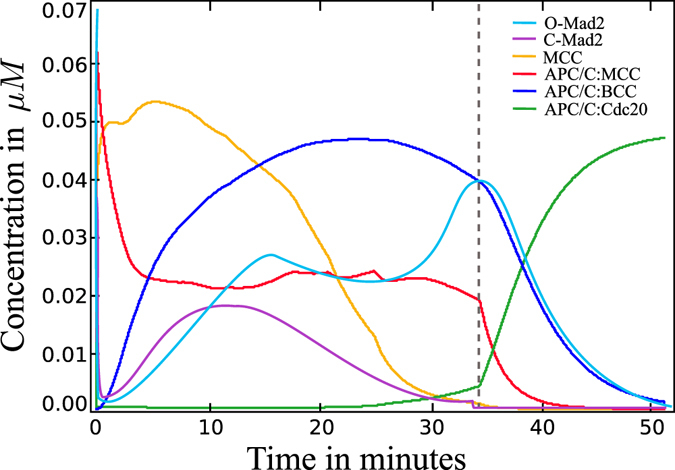
Wild-type concentration over time for the labeled species. The dashed line indicates attachment of the last kinetochore. The MCC is produced quickly and decays continuously. APC/C:MCC rapidly captures and disables APC/C molecules. Apc/C:MCC and APC:Cdc20:MCC yield the less stable APC/C:BCC. APC/C:Cdc20 is present during the whole process and reaches its full activity in the period of the last attachment.

Nevertheless, we see fast switching between disabled and activated APC/C taking place a few minutes after the last proper attachment (cf. Fig. 3). This results in a peak of Securin degradation, as measured by Dick *et al*.^11^.

### Particle simulation emulates the rheostat switch and reveals the minor role of space

Results from the particle simulation look qualitatively similar to the outcome of the ODE simulation (cf. Fig. 3). The anticipated switching behavior can be seen, although the transition from silenced to activated APC/C is not as sharp as the one found in the ODE simulation. Seeing as kinetochores emit p31^comet^, which has a high diffusion coefficient, the silencing ought to be quite fast but we did not model p31^comet^ molecules explicitly, only via an increased interaction radius of attached kinetochores. We used this simplification for performance reasons, as the concentration of p31^comet^ is high^27^ and its simulation would require a lot of computational power.

APC/C:Cdc20 is present and active, supporting the rheostat switch in Securin concentration. Aside from fluctuations, which result from the stochasticity of the simulation, all species concentrations are similar to those obtained in the ODE simulation. The setup of our simulation, described in the methods section, leads to the hypothesis that diffusion and reaction rates, as well as the extension of all species, do not play major roles in the functioning of the SAC. As pointed out in the previous section, the kinetochore-allocated reaction does play a major role. In our model, any O-Mad2 particle that enters a kinetochore region is converted into C-Mad2, which makes this reaction only space- and diffusion-dependent. Accordingly, proper SAC functioning requires that proteins be concentrated around the kinetochore plate (cf. Fig. S2). Other spatial properties, such as diffusion and reaction rates, as well as the extensions of the proteins, only play minor roles.

### Mutant experiments validate the behavior of the system

Several studies reported the influence of crucial protein mutations on the qualitative behavior of the SAC^8, 28–33^. These include the depletion and over-expression of the core components Mad2, BubR1:Bub3 and Cdc20. These studies help to validate our model, as they show how the artificial system responds to changes in the initial conditions. We modeled depletion by reducing the initial concentrations (cf. Table S2) to 5% and 40% of their original values, and modeled over-expression by a tenfold increase over the original initial concentration.

The *in*-*silico* mutation experiments (cf. Fig. S3) were conducted with the following configurations: (**FI**) full inhibition: low APC/C:Cdc20 concentration during attachment; (**WI**) weak inhibition: medium APC/C:Cdc20 concentration during attachment; (**NI**) no inhibition: high APC/C:Cdc20 concentration during attachment; (**PE**) proper exit: fast reactivation of APC/C:Cdc20; (**DE**) delayed exit: slow reactivation of APC/C:Cdc20; and (**IE**) improper exit: no reactivation of APC/C:Cdc20 or premature reactivation of APC/C:Cdc20.

**Figure 4:**
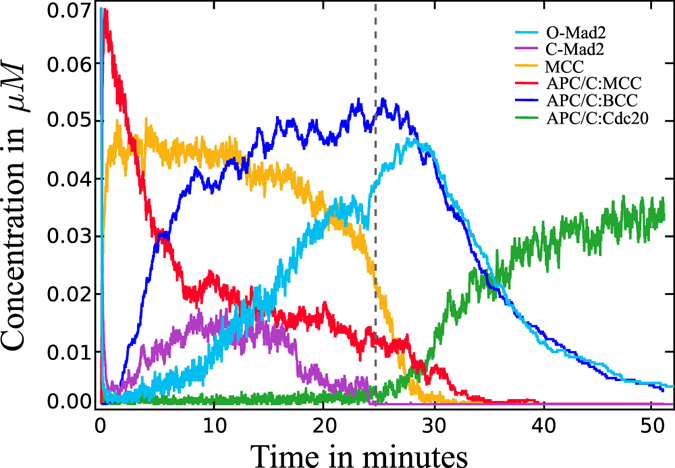
Spatial concentration over time for the labeled species. The dashed line indicates attachment of the last kinetochore. The mainly difference to the ODE curves is the slower activation of APC/C:Cdc20 and the presence of fluctuations in all species.

These classifications are sufficient for gaining a qualitative insight into the disturbed system. We only used the ODE model for these studies, given that its behavior was the same as the particle system (cf. Figs 3 and 4).

Without any initial mutations, our model behaves as reported in several studies (notated as wild-type in Table 1).

**Table 1:** Comparison of the *in*-*vitro* and *in*-*silico* mutation experiments.

Type of mutation	Experiment	*in*-*silico*	*in*-*vitro*	Remarks
Wild-Type	—	(FI) - (PE)	(FI) - (PE)	8
Mad2	O	(FI) - (PE)	(FI)	28
	D 5%	(NI) - (IE)	(NI)	29
	D 40%	(NI) - (IE)	(NI)	10
BubR1:Bub3	O	(FI) - (PE)	(FI)	30
	D 5%	(NI) - (IE)	(NI)	31
	D 40%	(FI) - (PE)	—	
Cdc20	O	(FI) - (PE)	(NI)	33
	D 5%	(FI) - (DE)	(FI)	32
	D 40%	(WI) - (DE)	—	

The comparison shows that all mutations follow the corresponding studies, aside from the over-expression of Cdc20. As the mechanism for how Cdc20 over-expression leads to a premature anaphase is not well understood^33^, and we used only simplest kinetics in our model, we are unable to reproduce these results. We suggest three approaches for achieving a SAC defect with over-expressed Cdc20: (1) create a competitive binding between MCC and Cdc20 to APC/C, so that MCC cannot target Cdc20-activated APC/C; (2) define kinetics for the binding to APC/C that favor Cdc20 over MCC if a large amount of Cdc20 is available; (3) define a new reaction rule so that Cdc20 silences MCC, i.e. MCC + Cdc20 → MCC:Cdc20. However, as these are purely speculative, we did not incorporate them in our model. In addition to the mutations mentioned in the literature, we also performed 40% depletion experiments on Cdc20 and BubR1:Bub3. Our study shows the crucial role of Mad2 as a reduction to 40% is sufficient for SAC dysfunction. On the other hand, BubR1:Bub3 can be reduced but still provide enough BCC to keep the APC/C inactive during metaphase.

### Switch analysis of the full model supports the rheostat hypothesis

**Figure 5:**
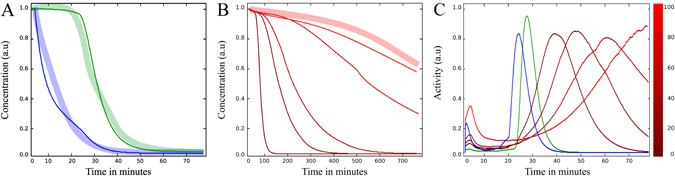
Securin behavior under different treatments. Thin lines are simulation data. Thick lines represent data points from Dick *et al*. Fig. 5 ^11^. The colorbar on the right represents the concentration of nocodazole in ng ml^−1^. (**A**) Total concentration of Securin over time, normalized at prometaphase onset (time = 0). The green curve shows the unperturbed system, in which Securin starts degrading at a significantly higher rate once kinetochores start attaching. The blue curve shows the influence of Mad2 targeting siRNA, simulated through the reduction of the Mad2 initial concentration. Here, APC/C:Cdc20 is not captured by MCC and Securin starts degrading immediately. (**B**) The same as in A, but under the influence of different concentrations of nocodazole, modeled by a stepwise decrease of the rate *k*_*attach*_. Curves are shown for concentrations in the range 1–100 ng ml^−1^ nocodazole. (**C**) The degradation rate of Securin (control in green, targeting siRNA in blue and nocodazole in red). As the rate peaks briefly before anaphase onset, these curves demonstrate how different drugs can arrest the cell in metaphase and delay anaphase.

We incorporated the decay of Securin into our model and found that it responds in the same way as the experimental data suggests (cf. Fig. 5). Securin is degraded continuously based on the level of MCC. Nocodazole and siRNA influence the degradation rate and the time of anaphase onset (cf. Fig. 5C). Given this outcome, our model supports the rheostat switch hypothesis with Securin.

In regulatory networks, the generation of an all-or-nothing toggle switch is achieved through the construction of a feedback loop that leads to bistability^34, 35^. Determining whether a system is bistable can be done via a bifurcation analysis. In our network, the feedback loop could be the dissembling of APC/C:MCC by APC/C, in which APC/C ubiquitinates Cdc20, decreasing its affinity to Mad2, and frees itself from the MCC. However, the incorporation of this feedback loop into the full model would be speculative as there are no kinetic data on this mechanism. For this reason we restrict our model to the core SAC mechanism in what follows.

### Switch analysis of the coarse-grained model reveals putative cooperation between the rheostat and toggle switch

**Figure 6:**
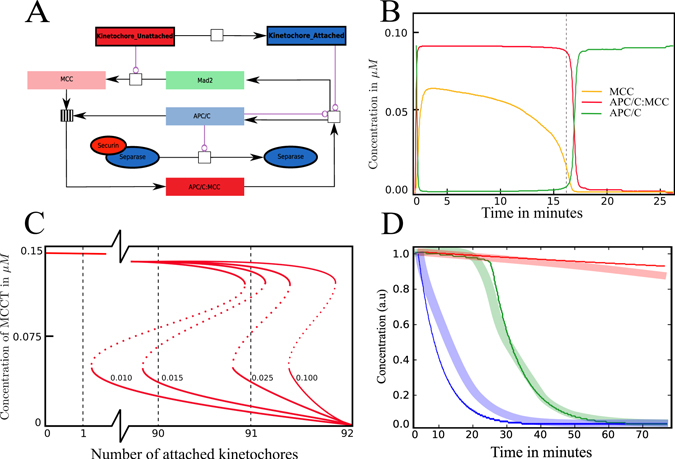
Switch analysis performed using the minimal model. (**A**) Reaction network for the crucial switching part (following the labeling conventions Fig. 2). (**B**) The concentration plot for core species over time showing the switch occurring after proper spindle attachment (dashed line, cf. Fig. 3). (**C**) One-parameter bifurcation diagrams showing the activity of the MCC against the number of attached kinetochores using different rates *k*_*M*1_ (labeled next to the curves). With a properly chosen rate, the dashed red lines clearly show that all kinetochores have to be attached to completely silence the MCC. To reengage the SAC, kinetochores have to reattach. If the rate drops below the estimated threshold, the SAC is silenced before all kinetochores are attached. (**D**) Securin concentration under different treatments for *in*-*vitro* (thick lines) and *in*-*silico* (thin lines). The green curve shows the wild-type, blue shows the influence of Mad2 targeting siRNA and red shows the influence of nocodazole. In all situations, securin degrades steadily, validating the rheostat switch hypothesis.

Our reduced model (similar to the one proposed by Verdugo *et al*.^36^) includes the formation of MCC and APC/C:MCC, and the reactivation of APC/C (cf. Fig. 6A) in the following way.

20 $${{\rm{Kin}}}_{{\rm{U}}}\mathop{\rightharpoonup }\limits^{{k}_{attach}}{{\rm{Kin}}}_{{\rm{A}}}$$

21 $${\rm{Mad2}}\mathop{\rightharpoonup }\limits^{[Ki{n}_{U}]\times {k}_{M1}}{\rm{MCC}}$$

22 $${\rm{MCC}}+{\rm{APC}}/{\rm{C}}\underset{{k}_{-M2}}{\overset{{k}_{M2}}{\rightleftharpoons }}{\rm{APC}}/C:\text{MCC}$$

23 $${\rm{APC}}/C:\text{MCC}\mathop{\rightharpoonup }\limits^{[APC/C]\times {k}_{M3}}{\rm{APC}}/{\rm{C}}+{\rm{Mad2}}$$

24 $${\rm{APC}}/C:\text{Cdc20}+{\rm{Securin}}\mathop{\rightharpoonup }\limits^{{k}_{D}}{\rm{APC}}/C:\text{Cdc20}$$

All initial concentrations and reaction rates are identical with the full model where possible. If reactions in the reduced model differ from those in the full model, new rates have been introduced (cf. Table S3). The model is most sensitive to *k*_*M*1_ as this can lead to a defect in SAC functioning. The rate has been estimated to be 0.016*s* ^−1^, both experimentally^22, 37^ and theoretically (cf. Text S1). As shown in Fig. 6C, the SAC does not work properly if this rate is decreased.

The outcome of the simulated minimal model in Fig. 6B shows the switch between active and inactive APC/C as well as the steady degradation of MCC. Concentration plots of Securin under different treatments are shown in panel C and these coincide with the response found in the detailed model and with experimental findings (cf. Fig. 5 and ref. 11).

To demonstrate the bistability of our minimal feedback model we performed a one parameter bifurcation analysis. Evaluating the quantitative behavior requires the total concentrations of Mad2, MCC and APC/C, which we derived from Fig. 6A. The concentration of APC/C, written as [APCT] = [APC/C] + [APC/C:MCC], is constant; likewise, the concentration of C-Mad2, [MadT] = [C-Mad2] + [MCC] + [APC/C:MCC]. The amount of MCC is given by [MCCT] = [MCC] + [APC/C:MCC] and changes over time according to

25 $$\frac{d[\text{MCCT}]}{{\rm{d}}t}={k}_{3}Ki{n}_{U}([\text{MadT}]-[\text{MCCT}])-{k}_{8}Ki{n}_{A}[{\rm{APC}}/{\rm{C}}]\,[{\rm{APC}}/C:\text{MCC}])$$

The parameter under study is the number of unattached kinetochores *Kin*_*U*_ in the range 0 to 92. Substituting the constant concentrations [APCT] and [MadT] into the steady state condition for APC/C:MCC gives

26 $${k}_{2}[\text{MCC}]\,[\text{APC}]={k}_{-2}[{\rm{APC}}/C:\text{MCC}]+{k}_{8}[{\rm{APC}}/C:\text{MCC}]$$

which results in an equation for APC/C:MCC in the steady state:

27 $${\rm{B}}={k}_{m}+[\text{APCT}]+[\text{MCCT}]$$

28 $$[{\rm{APC}}/C:\text{MCC}]=\frac{{\rm{B}}-\sqrt{{\rm{B}}-4[\text{MCCT}]\,[\text{APCT}]}}{2}$$

where *k*_*m*_ is the relation (*k*_−2_ + *k*_8_)/*k*_2_. The bifurcation diagram in Fig. 6D shows the typical s-shaped curve of a bistable switch. It demonstrates clearly that all kinetochores have to be attached to disengage the SAC, while at least one must reattach to reengage the SAC.

This finding shows that it is possible to observe a continuous degradation of one species (Securin), leading to the assumption of a rheostat switch, while the underlying core components (MCC) behave like a toggle switch.

## Conclusion

The SAC is a crucial mechanism for guaranteeing correct DNA segregation. It achieves this through a robust switch in APC/C activity, but the details of how this switch works have remained elusive. In this work, we have presented the most detailed biochemical model of the human mitotic checkpoint that includes a SAC effector such as Securin. The model reproduces experimental mutation experiments qualitatively as well as recent studies of the rheostat behavior quantitatively. In addition to simulating the switch using classical ODEs, we also studied the SAC using a detailed spatial particle simulation that explicitly included all 92 kinetochores. This particle simulation suggested that diffusion rates and spatial arrangements of kinetochores are not important for explaining SAC function. However, a concentration threshold is needed and this is achieved through compartmentalization, as we only took the region close to the kinetochores in account. For this reason, a non-spatial ODE model is sufficient for elucidating SAC behavior. When we simulated different scenarios with various perturbations of the spindle apparatus (nocodazole and Mad2 targeting siRNA), our model reproduced the recently found rheostat behavior. Nocodazole was found to delay the anaphase onset, while siRNA was found to lead to a premature mitotic exit. Notably, the switch in our detailed model did not require a toggle switch. However, an autocatalytic feedback loop is postulated in the literature (APC/C and APC/C:MCC), and including this mechanism in a more abstract version of our model revealed a toggle switch in the core component (MCC) that did not prevent the output (Securin) behavior still appearing like a rheostat. So we conclude that observations on one species cannot determine all the details of the mechanism controlling the spindle assembly checkpoint. This suggests that mitotic control results from the interplay of several switches of different kinds, which cooperate to maintain the SAC and assure proper cell division.

## Methods

### Methods

#### Ordinary differential equation model

Setting up a system of differential equations requires all species and their reaction rules. A list of all reaction rates is provided in Table S2. Note that we used mass-action kinetics for all interactions, which can be expressed in the following general form:

29 $$\frac{d[Species]}{dt}={R}_{j}([Specie{s}_{i}];{P}_{j}).$$

This equation describes the behavior of a certain *Species* over time undergoing reactions *R*_*j*_, depending on other *Species*_*i*_ with the kinetic law *P*_*j*_.

#### Spatially-stochastic model

Particle simulations need spatial information in addition to reactions and laws. Necessary properties are shape, size, and the relative diffusion coefficients of every protein. Those characteristics have to be *wet*-*lab* estimated and are often not known. All proteins involved in the SAC are treated as spheres with homogeneous density. Given the molecular mass, which is generally known, we can calculate the radius of the relevant sphere and model its free diffusion in the cell (Text S2). The initial concentration of all species is converted into particle number via the reaction volume. These particles are then placed randomly in a reaction volume and undergo Brownian motion. Reaction rules are defined between species using reaction rates. If two particles hit each other and a rule has been defined between them, then the rate determines whether they react or not. First-order reactions (such a kinetochores attachment) happen spontaneously according to the corresponding reaction rate.

Spatial features like known barriers, shapes or restrictions of the reaction volume are realized with potentials. We model kinetochores as spherical particles. We also model the shape and size of the nuclear space during metaphase, and knowledge about the orientation of all chromosomes in the middle of the cell.

#### Numerical Calculations

ODE simulations were performed by the LSODA solver in Copasi^38^. This hybrid algorithm allows both particle and concentration treatments based on a user defined threshold (we choose the threshold to be 100). Kinetochores have only finite states (attached and unattached) so a Gillespian treatment is desired. All species outnumber the amount of kinetochores a thousandfold and thus are treated deterministically as concentrations.

Particle simulations were performed using the ReaDDy software^39^. Valid reactions and potentials are defined as well as general properties like simulation time and time-step size. To realize the simulation of around 20 system minutes, scaling of the time axis was essential, including a scaling of the reaction rates (Text S3).

The bifurcation analysis was done in XPPAUT^40^. Source code to all models is provided in the supplement material.

## Electronic supplementary material

Supplementary Information
