# Supplementary material for: A Dynamical Model for Activating and Silencing the Mitotic Checkpoint
Source: Sci Rep. 2017 Jun 20;7:3865. doi: 10.1038/s41598-017-04218-2 (PMC5478649; doi:10.1038/s41598-017-04218-2)
Supplement: Supplementary file 1 — Supplementary Information [file 41598_2017_4218_MOESM1_ESM.pdf]

A Dynamical Model for Activating and Silencing the Mitotic Checkpoint

Richard Henze[1], **Peter Dittrich**[1], **and Bashar Ibrahim**[1,2,*]

[1]Department of Mathematics and Computer Science, Friedrich Schiller University Jena, 07743 Jena, Germany
[2]Institute for Numerical Simulation, University of Bonn, 53115 Bonn, Germany
[*]bashar.ibrahim@uni-jena.de

ABSTRACT

Supplement Material

Text S1 Turnover rate at unattached kinetochores

We want to calculate the average time that a particle needs in order to enter a sub-volume in the reaction vessel (in our case, this is the time it takes for an O-Mad2 particle to hit an unattached kinetochore). The radii of the nucleus and kinetochore are R and r, respectively, and C-Mad2 diffuses with a coefficient D. Applying a spherical Taylor expansion to an average hitting time model, and applying Neumann boundary conditions, leads to the following formula:

$$\langle T \rangle \approx \frac{R^3}{3Dr} \tag{1}$$

We can use this formula to calculate how long it takes for all O-Mad2 particles to diffuse to a kinetochore. In particular, we can calculate the average hitting time, which is the time needed for half the particles to diffuse to a kinetochore before that time. It is the half-time of the following reaction:

$$\text{O-Mad2} \xrightarrow{k3\times[KinU]} \text{C-Mad2} \tag{2}$$

Using the relationship between the half-time and the reaction rate

$$k = \frac{ln(2)}{t_{1/2}} \tag{3}$$

we can estimate the maximum turnover rate of C-Mad at the kinetochores. Assuming a nucleus radius of $6\mu m$, a kinetochore radius of $0.1\mu m$ and the Mad2 diffusion coefficient of $16.61\mu m^2 s^{-1}$, we find a half-time of 43 s, which is consistent with experimental findings and results in a turnover rate of $0.016s^{-1}$ at each kinetochore.

Text S2 Calculating diffusion coefficients

In addition to the reactions and their governing laws, a particle simulation also needs spatial information. It requires knowledge of the shape and size of every protein and their relative diffusion coefficients. For the sake of simplicity, we model all proteins as spheres with uniform densities. Given the mass, which is known from lab-experiments, and assuming a uniform spherical particle, we can calculate the radius of the sphere using

$$r_i = 0.066 m_i^{1/3} \tag{4}$$

in which m_i refers to lab-measured mass of the molecule in Da and r_i is the radius of the molecule in nm. Following the Stokes-Einstein equation we can calculate the diffusion coefficient using

$$D_i = \frac{k_B T}{6\pi\eta r_i} \tag{5}$$

where

$$
\begin{aligned}
D_i &= \text{diffusion coefficient in } m^2 s^{-1} \\
k_B &= \text{Boltzmann-Constant } (1.380 \times 10^{-23}\text{J/K}) \\
T &= \text{temperature in K} \\
r_i &= \text{radius of the assumed spherical-particle in m} \\
\eta &= \text{viscosity of the medium in Ns/m}^2 (0.891 \times 10^{-3} \text{ in water;} \\
&\quad 6.75 \times 10^{-3} \text{ in nucleus})
\end{aligned}
$$

Text S3 Coarse-graining

Typical time-steps in particle simulations are 10^{-9}s, which would imply 1.2×10^{11} time-steps to realize the full metaphase (around 20 real-time minutes). Realistic particle numbers are around $1,000,000$ per species, which leads to an infeasible large time requirement for simulating the full system. For reference, simulating $1,751$ particles for a total of 8×10^7 time-steps takes around one week. For this reason, we introduce two coarse-graining techniques: (1) pseudo particles and (2) scaling of the time. One approach to reducing the amount of particles is to enlarge their reaction-surface and decrease their number. Every particle has a radius r_i and a surface where it interacts with other particles. As particles are assumed to be spherical, this surface is $\frac{4}{3}\pi r_i^2$. A number of particles can be merged to one bigger one:

$$
N_i \to \hat{N}_i, \text{with } N_i \gg \hat{N}_i \tag{6}
$$

$$
r_i \to \hat{r}_i, \text{with } r_i < \hat{r}_i \tag{7}
$$

where N_i is the number of particles of type i, $\hat{N}_i$ is the reduced number and $\hat{r}_i$ is the increased radius of pseudo-particle i. Under the constraint that the reaction surface must be conserved, the formula for the new radius is given by

$$
N_i 4\pi r_i^2 = \hat{N}_i 4\pi \hat{r}_i^2 \tag{8}
$$

$$
\Rightarrow \hat{r}_i = r_i \sqrt{\frac{N_i}{\hat{N}_i}} \tag{9}
$$

It is also possible to reduce the time in the same manner. A process taking time T can be reduced to $\hat{T}$ using a factor f_t so that

$$
T \to \hat{T} = f_t T, \text{ with } T \gg \hat{T} \tag{10}
$$

To ensure that all reactions reach the steady state in time $\hat{T}$ (if such a state exists), every reaction rate has to be multiplied by the factor f:

$$
k_i \to \hat{k}_i = f k_i \tag{11}
$$

In a reaction system with relevant spatial characteristics, the time-scaling means that a given particle may no longer be able to cover the distance d between point $P = (p_x, p_y, p_z)$ and $Q = (q_x, q_y, q_z)$. Thus, the diffusion coefficients for each type of particle must also be modified. In n-dimensional space the mean-square-displacement (MSD) is an estimate for the average distance a particle has moved and is calculated using

$$
MSD_i = 2dTD_i \tag{12}
$$

where

$$
\begin{aligned}
d &= \text{dimension of the simulation space} \\
T &= \text{simulated time} \\
D_i &= \text{diffusion-coefficient of particle i}
\end{aligned}
$$

The square root of the MSD is used as the mean distance that the particle has moved from its origin

$$dist_i = \sqrt{MSD_i} \tag{13}$$

and this $dist_i$ must also conserved through the time-scaling process:

$$\sqrt{2dTD_i} = dist_i = \sqrt{2d\hat{T}\hat{D}_i} \tag{14}$$
$$\hat{D}_i\hat{T} = TD_i \tag{15}$$
$$\hat{D}_i f_t T = TD_i \tag{16}$$
$$\hat{D}_i = f_t D_i \tag{17}$$

This theoretical result is valid given the limited range of diffusion coefficients of $\approx 0.5 - 30\mu m^2 s^{-1}$. If the compression-factor f exceeds 100 (which is quite a low factor) then the diffusion coefficient can no longer be adapted in this way, because the displacements of particles in each time-step would become large and the system would become unstable.

An alternative way to make the particle move from P to Q in the scaled time $\hat{T}$ is to decrease the distance between the points by scaling the space (the reaction volume). A space of dimensions X, Y, Z can be transformed using

$$S = (X,Y,Z) \rightarrow (\hat{X},\hat{Y},\hat{Z}) = \hat{S} \tag{18}$$

where every individual point is transformed according to

$$p = (x,y,z) \rightarrow f(p) = (\frac{x}{f_s}, \frac{y}{f_s}, \frac{z}{f_s}) \tag{19}$$

This transformation represents a consistent spatial dilation of all axis by an as yet unknown factor f_s. Given that dilatations are length-conserving, the distance in the new space is scaled directly by the factor f_s according to

$$d = \frac{\hat{d}}{f_s} \tag{20}$$

One distance of interest is the MSD (described above), which gives the average displacement of a particle by diffusion. The relation between this distance and the time-compression-factor is

$$\text{MSD} = \sqrt{6DT}$$
$$\hat{\text{MSD}} = \sqrt{6D\hat{T}}$$
$$\Rightarrow MSD = f_t \hat{\text{MSD}}$$
$$\Rightarrow d^2 = f_t \hat{d}^2$$

which can be incorporated into Equation (20) to give

$$f_s = \sqrt{f_t} \tag{21}$$

Those two scalings in time and space ensure that a particle which took δ seconds to move a distance η in the original system, needs $f_t\delta$ seconds to cover a distance from $f_s\eta$ in the scaled system.

References

1. Ibrahim, B., Diekmann, S., Schmitt, E. & Dittrich, P. In-silico modeling of the mitotic spindle assembly checkpoint. *PLoS ONE* **3** (2008).

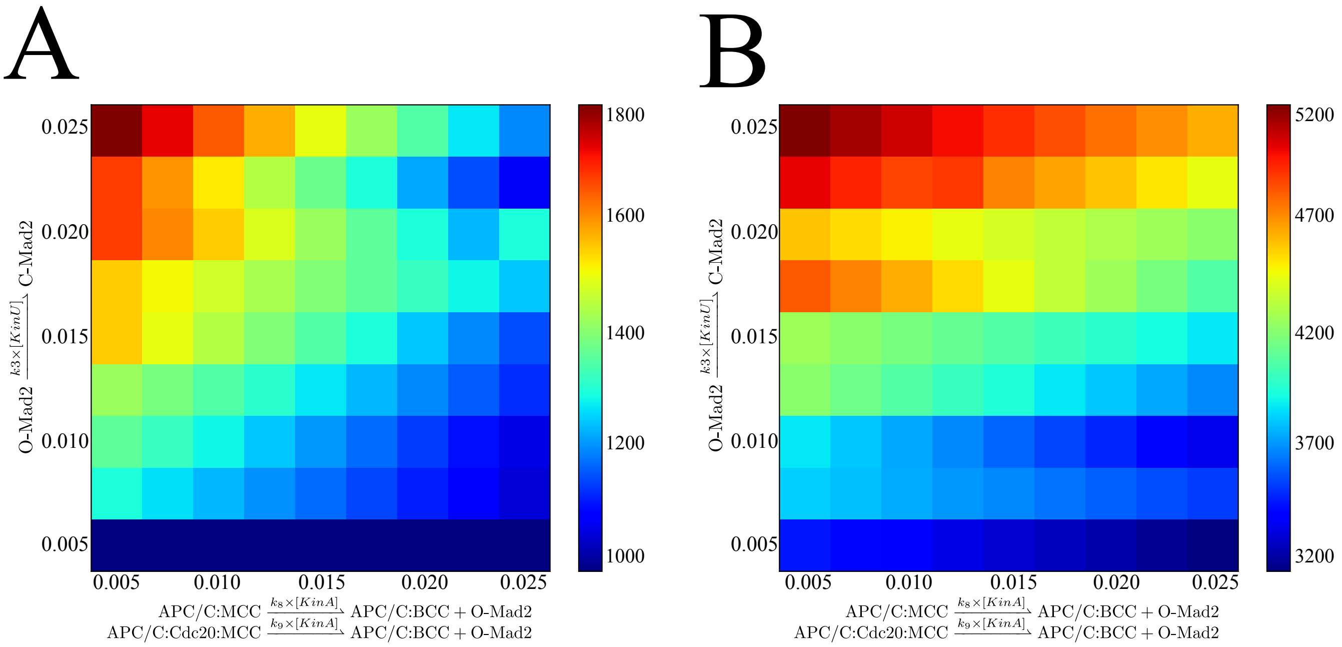

Figure S1. Parameter study for the most crucial reaction rates, that are k_3 and k_8. The colorbars on the right of each panel present the half-time of Securin degradation in seconds. The x-axis varies the rate of the labeled reaction (k_3) while the y-axis alters reaction rate k_8. **A:** Normal mitosis, where all kinetochores attach in an average time of $20-30$ minutes, corresponding to ≈ 1500 seconds. Looking at the presented heatmap this corresponds to a value of > 0.015 for k_3 and < 0.015 for k_8. **B:** Disturbed mitosis, where one chromosome is unable to attach. It shows that single kinetochores are able to extend metaphase for several hours (the shown values are only half-times of Securin). The rates for a reliable arrest in mitosis coincide with the ones in panel A. Both panels together suggest that k_3 value should be around 0.015 as it was theoretically determined to be a maximum of 0.016 (cf. Text S1) and k_8 should not exceed 0.015. In other terms have both kinetochores a strong enough influence to maintain the SAC as their reaction rates are similar.

2. Howell, B. J., Hoffman, D. B., Fang, G., Murray, A. W. & Salmon, E. D. Visualization of Mad2 dynamics at kinetochores, along spindle fibers, and at spindle poles in living cells. *J. Cell Biol.* **150**, 1233–1250 (2000).

3. Ibrahim, B., Dittrich, P., Diekmann, S. & Schmitt, E. Mad2 binding is not sufficient for complete cdc20 sequestering in mitotic transition control (an in silico study). *Biophysical Chemistry* **134**, 93–100 (2008).

4. Ibrahim, B., Schmitt, E., Dittrich, P. & Diekmann, S. In silico study of kinetochore control, amplification, and inhibition effects in mcc assembly. *BioSystems* **95**, 35–50 (2009).

5. Musacchio, A. & Salmon, E. D. The spindle-assembly checkpoint in space and time. *Nat. Rev. Mol. Cell Biol.* **8**, 379–393 (2007).

6. De Antoni, A. *et al.* The Mad1/Mad2 complex as a template for Mad2 activation in the spindle assembly checkpoint. *Curr. Biol.* **15**, 214–225 (2005).

7. Dick, A. E. & Gerlich, D. W. Kinetic framework of spindle assembly checkpoint signalling. *Nat. Cell Biol.* **15**, 1370–1377 (2013).

8. Fang, G. Checkpoint protein BubR1 acts synergistically with Mad2 to inhibit anaphase-promoting complex. *Mol. Biol. Cell* **13**, 755–766 (2002).

9. Tang, Z., Bharadwaj, R., Li, B. & Yu, H. Mad2-Independent inhibition of APCCdc20 by the mitotic checkpoint protein BubR1. *Dev. Cell* **1**, 227–237 (2001).

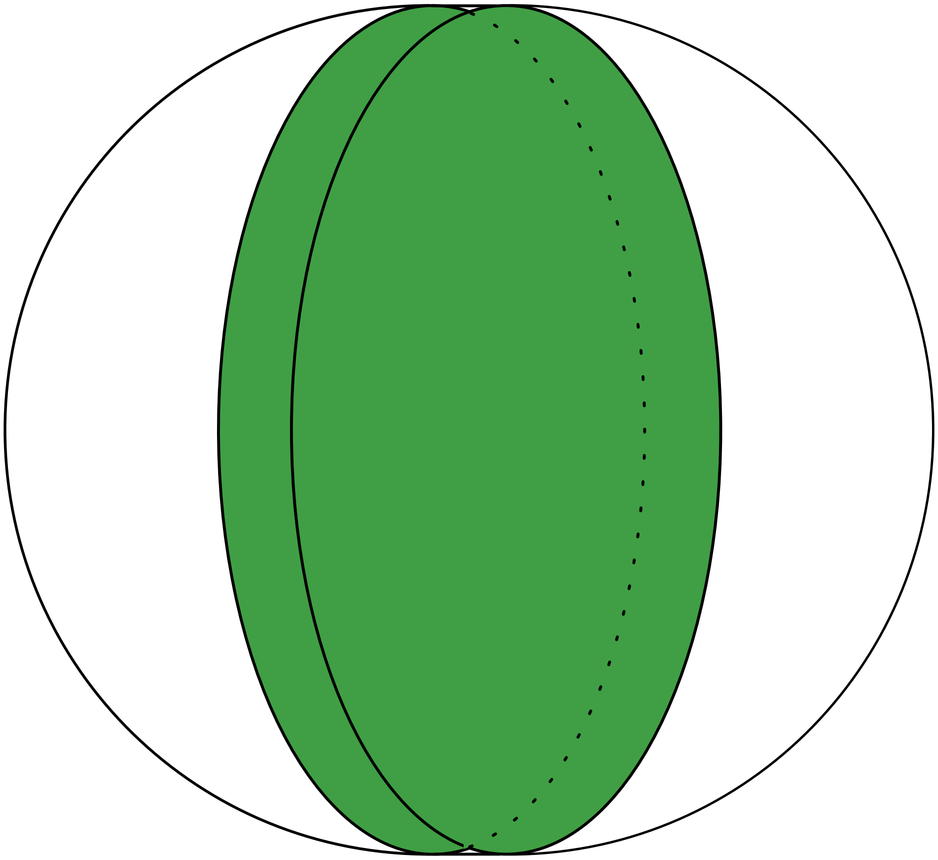

Figure S2. Shown is the schematic reaction volume used for the spatially-stochastic simulation. The whole nuclear space with a radius of $6\mu m$ is modeled, but all species are hold in the green area. This space exclusion is necessary to guarantee fast turnover from O-Mad2 to C-Mad2. All our model only take place in the green area, which can be seen as a well-mixed soup.

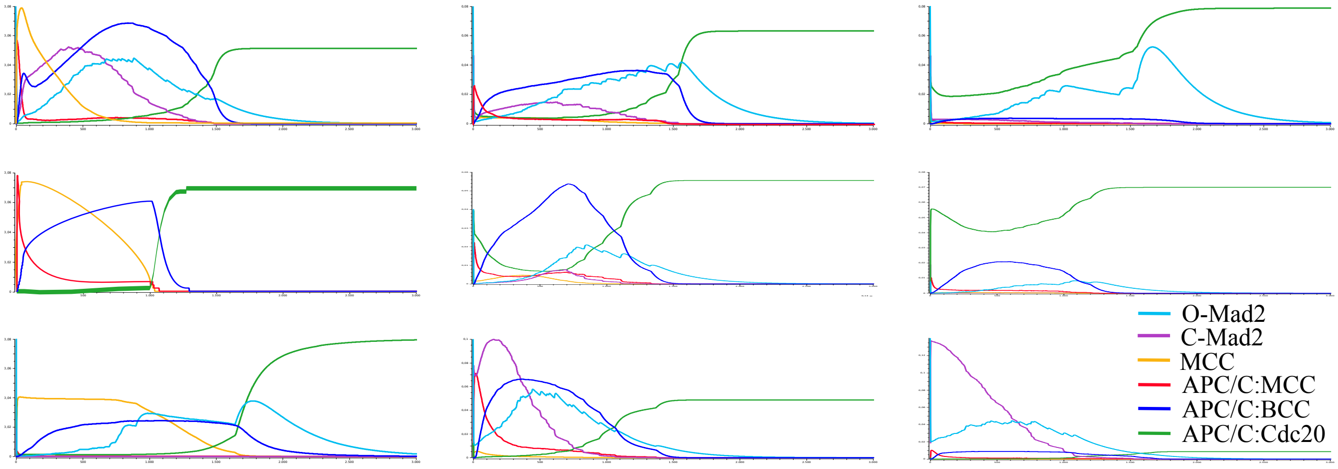

Figure S3. Simulation outcome of all mutation experiments. Rows correspond to the species BubR1:Bub3, Mad2 and Cdc20, respectively. Columns present an initial 10-fold over-expression, 40% depletion and 5% depletion, respectively. Axis and labels of the curves coincides with the ones in figure 3 and 4. Last kinetochores attachment is after 1500 seconds. Outcome of the classification can be found in the manuscript.

Table S1. **Kinetic Parameters of the full SAC model**

Classification	Parameter	Value	Remark
Attachment	k_{attach}	$0.0035 s^{-1}$	Metaphase around 20min
Activation			
	k_2	$100 \mu M^{-1} s^{-1}$	*in-silico* parameter study[1]
	k_{-2}	$0.08 s^{-1}$	*in-silico* parameter study[1]
	k_3	$> 0.015 s^{-1}$	per kinetochore; taken from Howell et. al.[2] and theoretical calculation
	k_{-3}	$0.2 s^{-1}$	taken from Howell et. al.[2]
	k_4	$10.0 \mu M^{-1} s^{-1}$	*in-silico* parameter study[3]
	k_5	$10.0 \mu M^{-1} s^{-1}$	*in-silico* parameter study[4]
	k_{-5}	$0.02 s^{-1}$	*in-silico* parameter study[4]
	k_6	$0.001 \mu M^{-1} s^{-1}$	taken from Musacchio[5]
	k_{-6}	$0.01 s^{-1}$	*in-silico* parameter study[3]
	k_7	$0.01 \mu M^{-1} s^{-1}$	*in-silico* parameter study[4]
	k_{-7}	$0.2 s^{-1}$	*in-silico* parameter study[4]
	k_{T1}	$0.01 \mu M^{-1} s^{-1}$	taken from DeAntoni et. al[6]
	k_{-T1}	$0.02 s^{-1}$	taken from DeAntoni et. al[6]
	k_{T2}	$10 \mu M^{-1} s^{-1}$	taken from DeAntoni et. al[6]
Silencing			
	k_1	$5 \mu M^{-1} s^{-1}$	*in-silico* parameter study[1]
	k_{-1}	$0.08 s^{-1}$	*in-silico* parameter study[1]
	k_8	$< 0.015 s^{-1}$	per kinetochore; this study
	k_9	$< 0.015 s^{-1}$	per kinetochore; this study
	k_{10}	$0.2 s^{-1}$	*in-silico* parameter study[4]
	k_D	$0.05 s^{-1}$	taken from Dick et. al[7]

Table S2. **Spatial Parameters of the SAC Model**

Species	Mass in kDa	Diffusion in $\mu m^2 s^{-1}$	Initial Concentration in μM	Source Concentration
O-Mad2	26.06	16.61	0.15	[2,8,9]
C-Mad2	26.06	16.61	0.01875	[2,8,9]
Cdc20	54.72	12.97	0.13	[8,9]
BubR1:Bub3	242.00	7.92	0.22	[8,9]
APC/C	836.5	5.23	0.09	[9]

Kinetochores initial amount is 92 and they do not diffuse. Other species particles start from zero. Their mass and diffusion coefficient combine from the basic blocks.

Table S3. **Kinetic Parameters of the coarse-grained SAC model**

Classification	Parameter	Value	Remark
Attachment	k_{attach}	$0.0035 s^{-1}$	Metaphase around 20min
SAC Activation			
	k_{M1}	$> 0.015 s^{-1}$	per kinetochore; taken from Howell et. al.[2] and theoretical calculation
	k_{M2}	$100 \mu M^{-1} s^{-1}$	*in-silico* parameter study[1]
	k_{-M2}	$0.08 s^{-1}$	*in-silico* parameter study[1]
SAC Silencing			
	k_{M3}	$< 0.015 s^{-1}$	per kinetochore; this study
	k_D	$0.05 s^{-1}$	taken from Dick et. al[7]
